# Items of General Interest

**Published:** 1915-08

**Authors:** 


### Items of General Interest.

J. A. Hammack, M. D., of Kennedale says that the waters of Kennedale contain medical properties which expel poison from the system and cure pellagra. He exhibited six patients, four children and two adults, who had been pellagra victims.

Contracts have been signed by the representatives of St. Paul's sanitarium. The contract will call for an expenditure of about $200,000. The wing will be built on the east side of the main building and will almost double the capacity of this well known institution.

Dr. Joseph Goldberger of the United States Public Health Service has come to Texas to investigate the pellagra conditions in Texas. The investigation of pellagra was begun in 1907 by the public health service. Last year Dr. Goldberger was made chief of this department and since then several stations for the treatment of this disease have been established in Georgia, the Carolinas, and at Jackson, Miss. About a year ago Dr. Goldberger made a trip to Texas to investigate the orphanages of Texas to observe pellagra cases, and on his present trip will make a more extended investigation.

Announcement was made that the new Dallas Polyclinic Post-graduate Medical School will probably begin work October 1. The officers are: President, Dr. John S. Turner; vice president, Dr. M. E. Taber; secretary, Dr. S. E. Milliken; treasurer, Dr. J. H. Smart; executive committeeman, Dr. W. C. Swain; publicity committee, Drs. M. P. Stone, Albert Wilkinson and A. H. Fortner. The selection of Dr. Turner as head of the new school met with the general approval of all concerned as he is the chairman of the medical committee of the Texas Medical Association to go to the American Medical Association convention.

Dr. D. E. F. Otis, delegate from the Dominican Republic to the Pan-American Medical Congress, said in a paper read by him at the convention that kisses spread tuberculosis. As a substitute, Dr. Otis suggested the pat-pat, which he claims is the accepted form in Dominica. The pat-pat Dr. Otis explained thus: When a couple, osculatorily inclined, meet, they should advance within handshaking distance and then, instead of grasping hands or kissing, pat each other on the cheek and smile.

Dr. Katherine B. Davis, commissioner of correction of the City of New York, says that in her fifteen years' experience with women delinquents she has never found a college girl among them. Dr. Davis insists that inefficient parents, and too little to eat are often responsible for the youthful criminal.

More than 5000 skulls of the early South American Indians have been collected by Dr. Joseph C. Thompson, who says: "The evidences of trepanning skill among the earliest Indians is shown by this exhibit for the first time. The surgical instruments consisted of sharpened flints of different shapes and sizes, and the collection shows that the first medicine men were no mean surgeons. Among the primitive people some very curious ceremonial rites take place. These rites at times assume the character of a vigorous massage to the body, and they even tapped the head of the dying man with a baton. This latter action might readily have resulted in lucky repair of the broken skull, thus giving them the first ideas of the operation."

Prof. W. W. Keen has established the Corinna Borden Keen Research Fellowship in the Jefferson Medical College, the income from which now amounts to $1000. The gift provides that the recipient of the fellowship shall spend at least one year in Europe, America or elsewhere (wherever he can obtain the best facilities for research in the line of work he shall select, after consultation with the faculty), and that he shall publish at least one paper embodying the results of his work as the "Corinna Borden Keen Research Fellow of the Jefferson Medical College." Applications stating the line of investigation which the candidate desires to follow shall be forwarded to Dr. Ross V. Patterson, sub-dean, Jefferson Medical College, Philadelphia, Pa.

The Moody Sanitarium of San Antonio, which has long been known as an ideal place for persons suffering from nervous and mental diseases, drug and alcohol addictions, and nervous invalids needing rest, becomes more and more popular each year. Only recently the owners have, again, made a number of improvements in the way of alterations and additions. These improvements will add very greatly to the capacity and efficiency of this institution. Physicians who have patients in need of especial care should feel no hesitancy in sending them to Moody's, knowing that they will receive every attention that can be given by an able, conscientious and painstaking staff of physicians and nurses.

